# High-resolution prediction of leaf onset date in Japan in the 21st century under the IPCC A1B scenario

**DOI:** 10.1002/ece3.575

**Published:** 2013-05-12

**Authors:** Mayumi Hadano, Kenlo Nishida Nasahara, Takeshi Motohka, Hibiki Muraoka Noda, Kazutaka Murakami, Masahiro Hosaka

**Affiliations:** 1 College of Agrobiological Resource Sciences, University of Tsukuba1-1-1 Tennoudai, Tsukuba, Ibaraki 305-8572, Japan; 2 Faculty of Life and Environment Sciences, University of Tsukuba1-1-1 Tennoudai, Tsukuba, Ibaraki 305-8572, Japan; 3 Japan Aerospace Exploration Agency2-1-1, Sengen, Tsukuba, Ibaraki 305-8505, Japan; 4 Meteorological Research Institute1-1 Nagamine, Tsukuba, Ibaraki 305-0052, Japan

**Keywords:** AGCM, global warming, green–red vegetation index (GRVI), phenology, remote sensing

## Abstract

Reports indicate that leaf onset (leaf flush) of deciduous trees in cool-temperate ecosystems is occurring earlier in the spring in response to global warming. In this study, we created two types of phenology models, one driven only by warmth (spring warming [SW] model) and another driven by both warmth and winter chilling (parallel chill [PC] model), to predict such phenomena in the Japanese Islands at high spatial resolution (500 m). We calibrated these models using leaf onset dates derived from satellite data (Terra/MODIS) and *in situ* temperature data derived from a dense network of ground stations Automated Meteorological Data Acquisition System. We ran the model using future climate predictions created by the Japanese Meteorological Agency's MRI-AGCM3.1S model. In comparison to the first decade of the 2000s, our results predict that the date of leaf onset in the 2030s will advance by an average of 12 days under the SW model and 7 days under the PC model throughout the study area. The date of onset in the 2090s will advance by 26 days under the SW model and by 15 days under the PC model. The greatest impact will occur on Hokkaido (the northernmost island) and in the central mountains.

## Introduction

Phenology is the study of the timing and cues of seasonal phenomena related to animals and plants. One example is the spring leaf onset (leaf flush) of some deciduous trees, which has been reported to be occurring earlier in the spring in several places around the world (Walther et al. 2002; Matsumoto et al. 2003; Badeck et al. 2004). Timing of leaf onset determines the active period of leaf photosynthesis, respiration, and transpiration, which in turn influences primary production (Myneni et al. 1997; Menzel and Fabian 1999) and climate feedbacks (Sitch et al. 2003). Early leaf onset can lead to increased damage caused by late-spring frost (Cannel and Smith 1986). Thus, a shift in the timing of spring leaf onset is an important issue in the assessment of climatic impacts on forest ecosystems. Among the many phenological phenomena, this study focused on the timing of spring leaf onset for deciduous trees in the cool-temperate region of Japan.

Leaf onset can be predicted based on an empirical relationship between environmental conditions and the growth process of leaf buds. In general, the buds of cool-temperate deciduous trees flush (expand) in response to warmth during the spring (Perry 1971). The elucidation of this fact led to the degree-day (thermal time) concept, in which the cumulative temperature above a certain threshold is linearly related to the growth rate of buds (e.g., Richardson et al. 2006). Most degree-day approaches count a quantity called the “growing degree-day” (GDD), which is defined as the sum of the amount of daily average temperature above a threshold value during the winter and spring. The simplest degree-day approach assumes that the leaf flush starts when the GDD exceeds a critical value (GDD_C_), which is assumed to be a constant. Studies have shown, however, that the critical value also depends on the winter chilling conditions (Cannel and Smith 1983; Murray et al. 1989; Hunter and Lechowicz 1992; Kramer 1994a, 1995). In fact, leaf onset in some species occurs at a lower GDD if they have experienced a sufficiently low temperature to fulfill the plant's chilling requirement and break winter bud dormancy (Perry 1971; Landsberg 1974; Campbell and Sugano 1975).

There are several types of models based on the degree-day approach, with various levels of complexity. Comparative studies of those models have revealed that more complex models do not necessarily perform better than simple models (Hunter and Lechowicz 1992; Chuine et al. 1998). The generalized models (e.g., Chuine 2000) that combine many aspects of different schemes contain many parameters and thus require elaborate work and large data sets for calibration.

In general, the sensitivity of leaf phenology to climate depends on the species (e.g., Murray et al. 1989; Morin et al. 2009). Moreover, even within a species, the sensitivity of leaf onset timing to temperature can differ among populations that have adjusted to different climatic conditions (e.g., von Wuehlisch et al. 1995; Ducousso et al. 1996; Doi and Katano 2008). However, the degree of species dependence and the role of climatic adaptation in the sensitivity of leaf onset to temperature remain unclear.

Most previous predictive studies calibrated and validated the leaf onset models using *in situ* data from only a few field sites (Cannel and Smith 1986; Murray et al. 1989; Kramer 1994b). Such an approach is also common in regional and continental-scale analyses. For example, Morin et al. (2009) took this approach to predict leaf phenology in North America. They calibrated the model with *in situ* data from only three sites and applied the results to the entire continent. However, such an approach cannot account for the known variability of onset characteristics within a species in response to geographical heterogeneity.

The aim of this study was to predict changes in future leaf onset dates of deciduous trees in Japan in response to climate change, at high resolution. To achieve this goal, we used two phenology models to predict onset dates in the 2030s and 2090s. Because of the great biodiversity arising from the complex geographical conditions in Japan (particularly the presence of rugged mountains), this analysis must be done at sufficiently high spatial resolution to account for the variability of phenological sensitivity across the region. Therefore, we utilized satellite remote sensing data (Terra/MODIS) to calibrate the phenology model and used a numerical prediction of future climate with high spatial resolution (20 km) to drive the model. Our primary objectives were to elucidate how much the spring leaf onset will be advanced or delayed in Japan in response to climate change and the geographic patterns of the change.

## Materials and Methods

### Study area

The study area included the main Japanese Islands (Hokkaido, Honshu, Shikoku, and Kyushu) and their general vicinity (29°58′15″N to 46°04′30″N, 129°01′45″E to 147°58′30″E on the Japanese Geodetic Datum 2000). All the spatial analyses were performed using rectangular geographic coordinates (a so-called lat-lon projection).

### Temperature data

**Figure 1:**
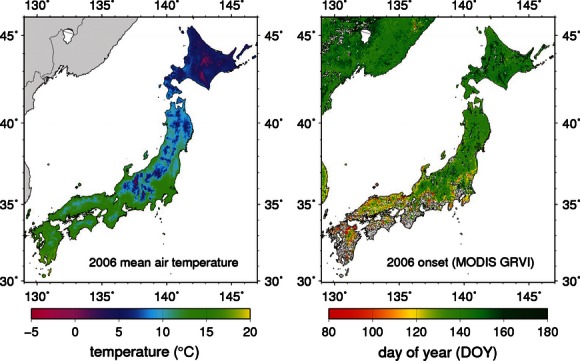
Left: Mean annual air temperature in 2006 derived from the ground weather network AMeDAS. Right: Leaf onset date in 2006, as derived using the green–red vegetation index (GRVI) method (Motohka et al. 2010) based on Terra/MODIS data at 500-m resolution. The areas without color are places where leaf onset was not detected.

We used two types of temperature data sets to drive the phenology model: past temperature data for calibrating the model and future climate-change scenario data for predicting the temperatures that would affect future leaf onset dates. The past data (daily mean air temperature from 1994 to 2009) were acquired by the Automated Meteorological Data Acquisition System (AMeDAS), which is managed by the Japan Meteorological Agency. In Japan, there are ∼1300 weather stations in the AMeDAS network. They are installed at ∼20-km intervals across Japan. We interpolated the station data using the following procedure. First, we converted the temperature at each site to the equivalent temperature at sea level by adding a bias of 0.6 K for every 100 m of elevation. Second, we interpolated the equivalent temperature at all the sites using a method based on regularized splines with tension (Mitášová and Mitáš 1993) and projected the results onto a 30-arc-second grid (i.e., a resolution of about 1 km). Finally, we converted the interpolated map of equivalent temperature to the real temperature at each elevation by subtracting the bias. This process was performed on data for each day during the entire period. The elevation data used for this process were taken from the U.S. Geological Survey GTOPO30 (http://eros.usgs.gov/#/Find_Data/Products_and_Data_Available/GTOPO30). Figure 1 (left) shows the mean temperature map for 2006 as an example of the temperature distribution derived from these data.

The future data set was obtained from a simulation made using the Japanese Meteorological Agency's MRI-AGCM3.1S model (Mizuta et al. 2006, 2008). The data set, which is called MRI-AM20km, contains daily average temperature values at 11′15″ resolution (about 20 km) throughout the Japanese Islands for three periods: the present (1979–2003), the near future (2015–2039), and the end of the 21st century (2075–2099). The simulation is based on the A1B emission scenario of the IPCC Special Report on Emission Scenarios (SRES; IPCC 2000). Generally speaking, the higher the spatial resolution of the model, the more realistically it accounts for topographic influences. The 20-km resolution is thought to be the current “best effort” for this scale of regional prediction. We extracted and utilized predictions for three decades from this data set, namely 1994–2003, 2030–2039, and 2090–2099. We disaggregated these data to a 30-arc-second grid (about 1 km) using the following procedure. First, we calculated the difference between the daily temperature maps derived from AMeDAS and MRI-AM20km during the present period, from 1994 to 2003. We then added this difference to the daily data for the two future periods (2030–2039 and 2090–2099).

### Phenology data

We utilized leaf onset maps derived from the MODIS sensor on the Terra satellite from 2001 to 2009 to calibrate the phenology models. The maps were derived using the algorithm of Motohka et al. (2010), which is based on the green–red vegetation index (GRVI). The MODIS data were the MOD09 A1 product (Surface Reflectance 8-Day L3 Global 500 m SIN Grid V004; 500-m resolution; 8-day composite). Many previous studies used the normalized difference vegetation index (NDVI) for the detection of leaf onset, but GRVI is better than NDVI in several aspects, especially its robustness in the face of errors caused by snow cover (Motohka et al. 2010; Nagai et al. 2010). The leaf onset date was assumed to correspond to the date on which GRVI exceeded 0.05 for the first time before day of year (DOY) 200. If this condition was not met at a pixel, which most often happened in evergreen forests, we assumed that leaf onset did not happen for the entire year at that pixel. Figure 1 (right) shows an example of a leaf onset map derived by this analysis.

### Phenology models

**Figure 2:**
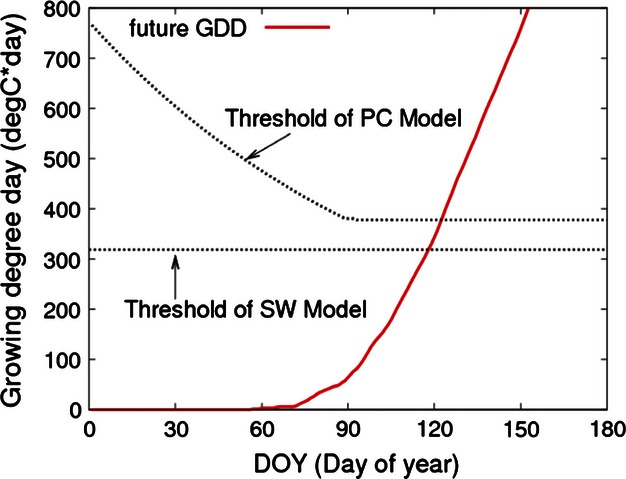
A conceptual diagram of the SW and PC models that we used to predict future leaf onset dates.

We mainly focused on the temperate and cool-temperate forests in Japan. As mentioned earlier, deciduous trees in cool-temperate ecosystems begin their bud expansion after exposure to a combination of chilling in winter and warmth in spring. However, the underlying mechanisms and formulation of the chilling effect are still unknown for many species and many circumstances. Therefore, we decided to employ two types of simple phenology model. The spring warming (SW) model is driven by warmth alone, whereas the parallel chill (PC) model is driven not only by warmth but also strongly by chilling. These two types of model were compared by Hunter and Lechowicz (1992). Figure 2 shows a conceptual diagram of the SW and PC models. Because these two models have the smallest (SW) and largest (PC) sensitivity to the chilling effect, which would be the dominant uncertainty factor controlling the spring phenology in this region, we assumed that the reality would lie between these two extreme models and that our analysis would be valid between these extremes.

The SW model is the simplest implementation of the degree-day approach. It calculates GDD as follows:


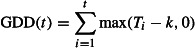
(1)

where *t* is time (DOY), *T*_*i*_ is daily mean air temperature on DOY *i*, and *k* is the minimum temperature required for growth. Although the starting DOY for counting GDD can be treated as a site-dependent variable, we assumed it to be 1 (1 January), which is a constant value for all regions. In future research, it would be worthwhile determining whether regional calibration of this starting day would improve the prediction accuracy. Although *k* may depend on both region and species, we assumed that *k* = 0°C, as this was the optimal value used for cool-temperate deciduous broadleaved forests in the analysis by Botta et al. (2000). We assumed that leaf onset occurred when GDD reached a critical (threshold) value (GDD_C_), which depends on the species and region but remains constant among years. We calculated GDD_C_ for each pixel for each year from 2001 to 2009 using the satellite leaf onset maps and the past temperature maps. Then, we calculated an interannual average of GDD_C_ for each pixel and used these values for the prediction of future leaf onset dates.

The PC model is similar to the SW model, but GDD_C_ also depends on winter chilling. It assumes that an increase in the number of chilling days (NCD) reduces a plant's requirement for spring warmth to initiate bud growth (Cannel and Smith 1983; Murray et al. 1989) and hence reduces GDD_C_. We used the method of Murray et al. (1989) and assumed GDD_C_ to be a function of the NCD, which is defined as follows:


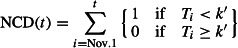
(2)

where *k′* is the maximum temperature at which the chilling effect occurs. We assumed *k′* = 5°C based on the analysis of Murray et al. (1989). We chose 1 November as the start of the chilling period based on the fact that most of the deciduous forests in this region complete leaf fall before this timing and the days with average temperature below 5°C increase after this timing. Leaf onset occurs when GDD reaches GDD_C_, which is an exponential function of NCD:


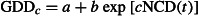
(3)

where *a* is the interannual mean of the difference between *b* exp[*c*NCD(*t*)] and GDD at the leaf onset date and *b* and *c* are constants that depend (in principle) on the species and region. In practice, we applied the parameters (*b* = 1084, *c* = −0.00904) for the group that was most sensitive to winter chilling (group 1) in the experiment of Murray et al. (1989) to all pixels. Using the satellite leaf onset maps and the past temperature maps, we adjusted *a* for each pixel.

### Future prediction

After calibrating the SW and PC models with the past climate data and satellite data (2001–2009), we ran the models to predict the future daily temperature time series for two periods (2030–2039 and 2090–2099) at a 15-arc-second (about 500 m) grid scale. As a result, we obtained a prediction of leaf onset date at each pixel in each year. We were unable to simulate regions where the phenology model could not be established (mainly because the satellite algorithm for phenology detection failed). We excluded these areas from our analysis.

To check the accuracy of the models, we ran the models for the past years using the following procedures. First, we excluded 1 year from 2001 to 2009 (i.e., leave-one-out validation) and then estimated the parameters of the models (GDD_C_ and *a*) with the aforementioned protocol using the remaining data. We considered the calibrated parameters to be independent of the data in that year. Next, we ran the model with those parameters for that year and compared the predicted onset date with the actual observed onset date. For example, we predicted the onset date in 2001 using data from 2002 to 2009, then compared that predicted date with the actual observed date in 2001. We carried out this process for every year from 2001 to 2009. As a result, we obtained the discrepancies between the model simulations and the observations for each year at each pixel. We calculated the RMSE (root-mean-square error) at each pixel and assumed that it represented the magnitude of the simulation error.

**Figure 3:**
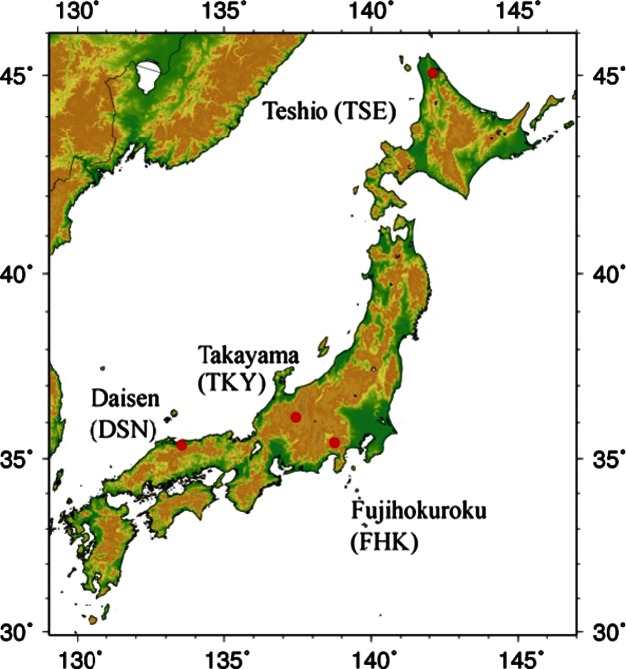
Map showing the four ground sites at which typical deciduous forests have been monitored regularly. These sites were investigated in more detail in our analysis.

We analyzed the entire region without selecting a particular land cover that has clear natural phenology (such as deciduous forest). Some parts of the study area may consist of agricultural land, grassland, or urban areas rather than deciduous forest, or may consist of a mixture of these types. The presence of a combination of multiple land covers would create mixed pixels. Therefore, some of what appear to be phenological signals in the analysis may instead be errors caused by other factors that arose from the complex landscape (such as changes in crop phenology due to differences in planting dates). To mitigate this problem, we studied pixels in more detail at four field study sites of deciduous forests using one pixel for each site: Teshio (TSE), Takayama (TKY), Fujihokuroku (FHK), and Daisen (DSN). These sites are considered to have spatial coverage bigger than the satellite pixel. Because the phenology in these sites are regularly monitored from the ground, we can expect that they will serve validation data in the future for the prediction made in this study. They are part of the AsiaFlux network of sites (http://asiaflux.net/), except for DSN, which belongs to the Internet Nature Information System (http://www.sizenken.biodic.go.jp/live/index.php), a monitoring network of automatic digital cameras maintained by the Ministry of Environment, Japan. Figure 3 shows the locations of these sites; Table 1 provides a detailed description of their key characteristics.

**Table 1:** The details of the four ground sites used in the detailed analysis

Teshio (TSE): conifer-hardwood mixed forest	
Location and elevation	45.051°N, 142.110°E, 70 m
Mean annual temperature Mean annual precipitation	5.7°C1000 mm
Dominant species	*Quercus crispula, Betula ermanii, Abies sachalinensis*
Takayama (TKY): deciduous broadleaved forest	
Location and elevation	36.146°N, 137.423°E, 1420 m
Mean annual temperature Mean annual precipitation	6.5°C2275 mm
Dominant species	*Quercus crispula, Betula ermanii*
Fujihokuroku (FHK): larch plantation	
Location and elevation	35.444°N, 138.765°E, 1100 m
Mean annual temperature Mean annual precipitation	9.6°C1566 mm
Dominant species	*Larix kaempferi*
Daisen (DSN): deciduous broadleaved forest	
Location and elevation	35.377°N, 133.540°E, 900 m
Mean annual temperature Mean annual precipitation	11.1°C2574 mm
Dominant species	*Quercus crispula, Fagus crenata*

## Results

The RMSE for the simulation was about 8 days in both models: on average, it was 8.0 days for the SW model and 8.2 days for the PC model. This is comparable to the temporal resolution of the satellite observations (8 days). Assuming that the error is independent among the years, an RMSE of 8 days for a single year at a single pixel would correspond to RMSE = 2.5 days (eight divided by the square root of 10 years) for the decadal mean at a single pixel.

**Figure 4:**
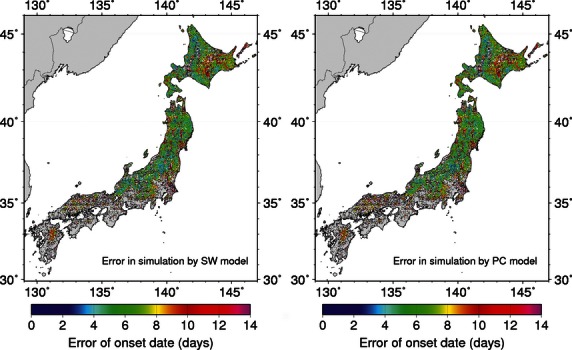
The distribution of the single-year prediction error (RMSE) for the leaf onset date for (left) the SW (spring warming) model and (right) the PC (parallel chill) model.

As is indicated by the spatial pattern of the errors (Fig. 4), many pixels had RMSE <8 days: 65% of the pixels for the SW model and 64% for the PC model. The largest errors (>10 days) were mainly for agricultural land. In addition, detection of the onset date failed in much of the southern and western parts of the study area, perhaps because those areas were mainly covered by evergreen trees. Therefore, the models appear to be most reliable in the central to northern parts of the study area, except for agricultural land.

**Figure 5:**
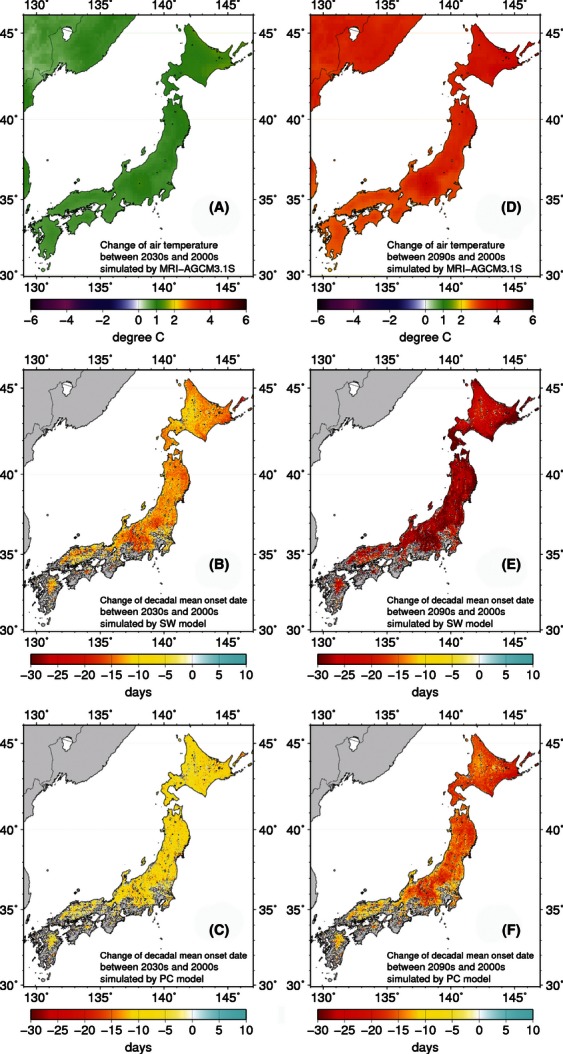
The influences of predicted climate change on the spring leaf onset data simulated by the SW and PC models. (A–C) Changes between the first decade of the 2000s and the 2030s (values are 2030s minus 2000s). (D–F) Changes between the first decade of the 2000s and the 2090s (values are 2090s minus 2000s). (A, D) Changes of the annual mean temperature predicted by the MRI-AGCM3.1S model under the IPCC A1B scenario. Positive values represent higher temperatures (warming) in the future. (B, E) Changes in the spring onset date predicted by the SW (spring warming) model. Negative values represent earlier onset in the future. (C, F) Changes in the spring onset date predicted by the PC (parallel chill) model. Negative values represent earlier onset in the future.

The simulations predicted an advance of the onset date in the future at most locations (Fig. 5B, C, E, and F). The magnitude of the advance was much greater using the SW model than using the PC model, probably because the PC model accounted for the decrease in the frequency of temperatures capable of breaking dormancy as a result of increasing temperatures (Fig. 5A and D).

In comparison with the first decade of the 2000s (2001–2009), the date of leaf onset in the 2030s will advance by about 12 days under the SW model (mean, 11.6 days; median, 12.5 days), versus about 7 days under the PC model (mean, 6.9 days; median, 7.6 days) throughout the study region (Fig. 5B and C). In other words, the advance will be by 2.3 days/decade under the PC model and 4.0 days/decade under the SW model. Both models predicted earlier onset in most of the study area. However, the SW model predicted earlier leaf onset than the PC model in most of the study area.

In comparison with the first decade of the 2000s, the date of leaf onset in the 2090s will advance by around 26 days under the SW model (mean, 26.0 days; median, 26.4 days) and by 15 days under the PC model (mean, 14.2 days; median, 15.2 days) throughout the study region (Fig. 5E and F). In other words, the onset date will advance by 1.6 days/decade under the PC model and 2.9 days/decade under the SW model. Both models predicted earlier onset in most of the study area, particularly on Hokkaido (the northern island) and in the central mountains.

**Figure 6:**
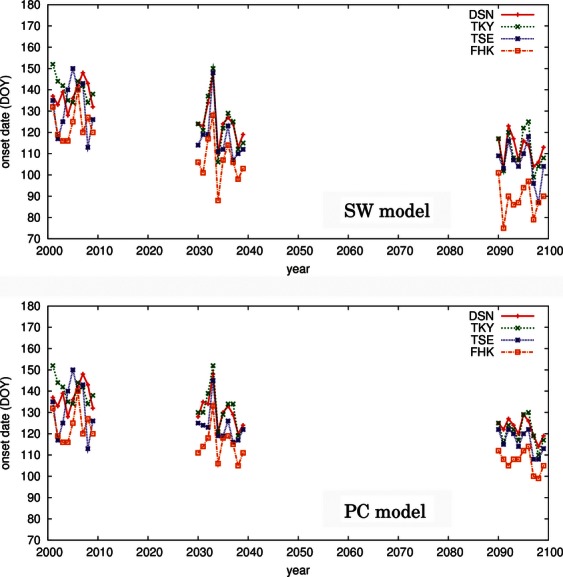
Changes in the leaf onset dates observed and predicted at the four sites that were analyzed in more detail: TSE, Teshio; TKY, Takayama; FHK, Fujihokuroku; DSN, Daisen. The locations and details of the sites are presented in Figure 3 and Table 1, respectively. The data for the period between 2000 and 2009 were obtained from satellite observations (MODIS GRVI). The rest of the data were obtained from the simulation using the two phenology models (SW, spring warming; PC, parallel chill).

Table 2 shows the adjusted model parameters for the four monitoring sites. The adjusted value of GDD_C_ was positively related to the mean annual temperature (Table 1) at the sites. Figure 6 shows the changes in leaf onset dates from the first decade of the 2000s to the 2030s and to the 2090s for the four sites. Both models predicted an advance of leaf onset at all four sites throughout the study period (until the 2030s and the 2090s). However, the magnitude of the changes differed somewhat; FHK showed a more sensitive response to future climate than the other sites. In addition, the PC model only predicted an advance in the leaf onset date at the site in the 2030s.

**Table 2:** The adjusted parameters for the leaf onset models at each site in Table 1

Site	GDD_C_ (°C-day)	*a* (°C-day)
Teshio (TSE)	215 ± 59	–20 ± 71
Takayama (TKY)	372 ± 77	117 ± 79
Fujihokuroku (FHK)	429 ± 82	86 ± 87
Daisen (DSN)	405 ± 53	124 ± 64

GDD_C_ was obtained using equation (1) for the SW model and *a* was obtained using equation (3) for the PC model. Both values were adjusted at each ground site. Values are the mean ± the standard deviation derived by leave-one-out approach.

## Discussion

### The advance of spring leaf onset

Both the SW model and the PC model predicted an advance of the leaf onset date. Because the SW model represents chilling-insensitive species and the PC model represents species with high chilling sensitivity, the reality is likely to be somewhere between these two models. The rate of change in the leaf onset date from the present to the end of the 2090s is predicted to be 2.9 days/decade according to the SW model and 1.6 days/decade according to the PC model. These values are comparable to existing observations in Japan; for example, Doi and Katano (2008) reported a rate of 2.7 days/decade from 1953 to 2005, and Matsumoto et al. (2003) reported a rate of 0.89 days/decade from 1953 to 2000. Therefore, our results suggest that for most deciduous trees in Japan, the leaf onset date will shift to become progressively earlier for at least the next 100 years.

### Geographic patterns of the change in leaf onset date

Leaf onset is mainly controlled by temperature, but the impact of temperature is not necessarily simple. In a cold region, for example, there might be two situations. In the first case, if the increase of temperature is moderate, it would likely remain sufficiently cold that the number of days with a temperature above the threshold (0°C for GDD and 5°C for NCD) would not increase greatly even under a warmer climate. Even if the temperature increased above the threshold, it would likely not be far beyond the threshold, hence the increment of GDD in spring would remain small. In this case, GDD and NCD would change little, as would the leaf onset date. This may be why the spatial pattern of the change of onset in the 2030s predicted by the PC model (Fig. 5C) shows a smaller and more homogeneous change than the pattern predicted by the SW model (Fig. 5B).

In the second case, if the increase of temperature is high or the present baseline temperature is already close to the threshold, a greater advance in the onset date may occur. This would be the case if the predicted temperature increase is particularly high in cold regions. This may be why the change pattern in the 2090s predicted by the PC model (Fig. 5F) corresponded well to the change pattern of temperature (Fig. 5D), particularly in Hokkaido (the northern island) and in the central mountains. In fact, JMA (2008) has predicted that a winter warming trend will be particularly evident at high latitudes in Japan during the 21st century. Therefore, in this second case, the spatial pattern of the onset date in Japan will change, particularly in cold regions.

### Primary factors underlying the changes predicted at the long-term monitoring sites

Among the four sites, the largest change was predicted at the FHK site, which belongs to the central mountains, under the SW model: leaf onset will occur 12.9 days earlier in the 2030s and 26.1 days earlier in the 2090s compared with values for the first decade of the 2000s. The present baseline temperature at FHK is relatively high, so a slight increase in temperature would result in more days being warmer than the threshold, such that GDD would start to increase earlier in the winter. This is a typical situation for the second scenario discussed in the previous section. Although the advance is smaller under the PC model than under the SW model, the PC model nonetheless predicted an advance of the onset date at all four sites. This indicated that a decrease in chilling opportunities would not outweigh the increase of GDD.

### Parameterization of the models

Because the goal of this study was to predict changes in the future onset date by establishing a potential range of uncertainty values, detailed analysis of the model's sensitivity to specific factors (e.g., a pixelwise analysis) is outside the scope of our study. Moreover, the detection of leaf onset using a satellite vegetation index such as GRVI is, at least in principle, site dependent. Because the index is influenced by site-dependent factors such as the solar angle (which depends on latitude), the background soil, the mixture of land cover types, and other factors, it would not be meaningful to directly compare the parameters and onset dates detected by this approach unless the sites locate in similar conditions. Therefore, we have focused only on the change of the onset date at each site. However, to some extent, the model parameters should be related to the acclimation mechanisms of the trees. For example, the positive correlation between GDD_C_ and the annual mean temperature was found (*r* = 0.62), suggesting that the tree species show at least some acclimation to the local climate. On the other hand, the decreased accuracy of the prediction in the warm regions (southwestern Japan) could be a consequence of our assumption that the starting date for calculating GDD could be set to a constant value (1 January) in equation (1). Investigating the effects of adjusting this parameter would require a pixelwise parameterization rather than the use of a regional-scale constant value for the parameter. Unfortunately, we could not perform this analysis because of limitations on the available data (particularly satellite data). This constraint could be mitigated in the near future by extending the spring phenology records in the satellite data.

### Limitations of this analysis

Clearly, uncertainties may exist in our results because of factors such as the presence of mixed-species forests, subpixel heterogeneity that was not detected by our analysis, differences in the degree of acclimation of different species, and human interventions in forest management. Satellite-based pixelwise phenology models, such as the ones used in this study, cannot attribute the results for a pixel to individual species unless the pixel is covered by a single species (which rarely happens in a landscape as variable as that of Japan). Therefore, instead of a complex model that takes species-level features into account, it is legitimate to make a range of predictions using two or more simple models. However, if most of the dominant species in a certain region have similar phenological features, and the features can be clearly identified and described, more accurate predictions could be obtained using a single model that accurately represents the common features in that region. However, the analysis is unlikely to be so simple in reality. For example, a transplanting experiment in Japan by Nunokawa and Tsukahara (2005) showed that the leaf onset date of Japanese beech (*Fagus crenata*) was earlier if the plants were obtained from a colder region and planted in a warmer region. In contrast, the onset date of *Betula ermanii* was later if it was taken from a region with a colder May temperature and planted in a warmer region (Ubukata 2003). These contrasts between species that often inhabit the same cool-temperate ecosystems in Japan would complicate analyses of the response of phenology to climate change at the ecosystem level.

Another limitation is related to the dynamics of land use and cover change. We did not account for temporal changes in species (e.g., succession) or in land use during the 21st century, and these changes would clearly affect the predicted changes in leaf onset date.

Finally, we used the climate predictions provided by a single model under a single SRES scenario. Future studies should examine the uncertainty of these results using multiple climate prediction models under a range of SRES scenarios.

## References

[b1] Badeck F-W, Bondeau A, Böttcher K, Doktor D, Lucht W, Schaber J (2004). Responses of spring phenology to climate change. New Phytol.

[b2] Botta A, Viovy N, Ciais P, Friedlingstein P, Monfray P (2000). A global prognostic scheme of leaf onset using satellite data. Glob. Change Biol.

[b3] Campbell RK, Sugano AI (1975). Phenology of budburst in Douglas-fir related to provenance, photoperiod, chilling and flushing temperature. Bot. Gaz.

[b4] Cannel MJR, Smith RI (1983). Thermal time, chill days and prediction of budburst in *Picea sitchensis*. J. Appl. Ecol.

[b5] Cannel MJR, Smith RI (1986). Climatic warming, spring budburst and frost damage on trees. J. Appl. Ecol.

[b6] Chuine I (2000). A unified model for the budburst of trees. J. Theor. Biol.

[b7] Chuine I, Cour P, Rousseau DD (1998). Fitting models predicting dates of flowering of temperate-zone trees using simulated annealing. Plant Cell Environ.

[b8] Doi H, Katano I (2008). Phenological timings of leaf budburst with climate change in Japan. Agric. For. Meteorol.

[b9] Ducousso A, Guyon JP, Kremér A (1996). Latitudinal and altitudinal variation of bud burst in western populations of sessile oak (*Quercus petraea* (Matt.) Liebl.). Ann. For. Sci.

[b10] Hunter AF, Lechowicz MJ (1992). Predicting the timing of budburst in temperate trees. J. Appl. Ecol.

[b11] IPCC (2000). Emission scenarios. Special Reports.

[b12] JMA (2008). Global warming projection, 7.

[b13] Kramer K (1994a). A modelling analysis of the effect of climatic warming on the probability of spring frost damage to tree species in the Netherlands and Germany. Plant Cell Environ.

[b14] Kramer K (1994b). Selecting a model to predict the onset of growth of *Fagus sylvatica*. J. Appl. Ecol.

[b15] Kramer K (1995). Phenotypic plasticity of the phenology of seven European tree species in relation to climatic warming. Plant Cell Environ.

[b16] Landsberg JJ (1974). Apple fruit bud development and growth: analysis and an empirical model. Ann. Bot.

[b17] Matsumoto K, Ohta T, Irasawa O, Nakamura T (2003). Climate change and extension of the *Ginkgo biloba* L. growing season in Japan. Glob. Change Biol.

[b18] Menzel A, Fabian P (1999). Growing season extended in Europe. Nature.

[b19] Mitášová H, Mitáš L (1993). Interpolation by regularized spline with tension: I. Theory and implementation. Math. Geol.

[b20] Mizuta R, Oouchi K, Yoshimura H, Yoshimura H, Noda A, Katayama K (2006). 20-km mesh global climate simulations using JMA-GSM model: mean climate states. J. Meteorol. Soc. Jpn.

[b21] Mizuta R, Adachi Y, Yukimoto S, Kusunoki K (2008). http://www.mri-jma.go.jp/Publish/Technical/DATA/VOL_56/tec_rep_mri_56.pdf.

[b22] Morin Z, Lechowicz MJ, Augspurger C, O'Keefe J, Viner D, Chuine I (2009). Leaf phenology in 22 North American tree species during the 21st century. Glob. Change Biol.

[b23] Motohka T, Nasahara KN, Oguma H, Tsuchida S (2010). Applicability of green-red vegetation index for remote sensing of vegetation phenology. Remote Sens.

[b24] Murray MB, Cannell GR, Smith RI (1989). Date of budburst of fifteen tree species in Britain following climatic warming. J. Appl. Ecol.

[b25] Myneni RB, Keeling CD, Tucher CJ, Asrar G, Nemani RR (1997). Increased plant growth in the northern high latitudes from 1981 to 1991. Nature.

[b26] Nagai S, Nasahara KN, Muraoka H, Akiyama T, Tsuchida S (2010). Field experiments to test the use of the normalized difference vegetation index for phenology detection. Agric. For. Meteorol.

[b27] Nunokawa K, Tsukahara M (2005). Leaf flush timing of *Fagus crenata* from different habitats. Bull. Niigata Prefect. For. Res. Inst.

[b28] Perry TO (1971). Dormancy of trees in winter. Science.

[b29] Richardson AD, Bailey AS, Denny EG, Martin CW, O'Keefe J (2006). Phenology of a northern hardwood forest canopy. Glob. Change Biol.

[b30] Sitch S, Smith B, Prentice IC, Arneth A, Bondeau A, Cramer W (2003). Evaluation of ecosystem dynamics, plant geography and terrestrial carbon cycling in the LPJ dynamic global vegetation model. Glob. Change Biol.

[b31] Ubukata M (2003). Ecological genetic study on the genetic resources and natural forest management of mizunara (*Quercus mongolica* var. *grosserrata*) in Hokkaido. Bull. For. Tree Breed. Cent.

[b32] Walther G-R, Post EP, Convey P, Menzel A, Parmesan C, Beebee TJC (2002). Ecological responses to recent climate change. Nature.

[b33] von Wuehlisch G, Krusche D, Muhs H-J (1995). Variation in temperature sum requirement for flushing of beech provenances. Silvae Genet.

